# Supplementary material for: Broca's cerebral asymmetry reflects gestural communication's lateralisation in monkeys (Papio anubis)
Source: eLife. 2022 Feb 2;11:e70521. doi: 10.7554/eLife.70521 (PMC8846582; doi:10.7554/eLife.70521)
Supplement: Transparent reporting form [file elife-70521-transrepform1.docx]

***eLife’s* transparent reporting form**

We encourage authors to provide detailed information *within their submission* to facilitate the interpretation and replication of experiments. Authors can upload supporting documentation to indicate the use of appropriate reporting guidelines for health-related research (see [EQUATOR Network](http://www.equator-network.org/%20)), life science research (see the [BioSharing Information Resource](https://biosharing.org/)), or the [ARRIVE guidelines](http://www.plosbiology.org/article/info:doi/10.1371/journal.pbio.1000412) for reporting work involving animal research. Where applicable, authors should refer to any relevant reporting standards documents in this form.

If you have any questions, please consult our Journal Policies and/or contact us: [editorial@elifesciences.org](mailto:editorial@elifesciences.org).

**Sample-size estimation**

- You should state whether an appropriate sample size was computed when the study was being designed
- You should state the statistical method of sample size computation and any required assumptions
- If no explicit power analysis was used, you should describe how you decided what sample (replicate) size (number) to use

Please outline where this information can be found within the submission (e.g., sections or figure legends), or explain why this information doesn’t apply to your submission:

Sample size was known, when the study was designed as both behaviour and brain images were coming from our exhaustive and growing MRI and behavioral databases. Although correlates between Inferior Arcuate sulcus depth asymmetry and handedness for gestural communication has never been done in primates before - which made challenging the determination of the precise effect size in babbons, 50 subjects was estimated as allowing robust statistical power based on previous significant results found in primate studies using the same sulci depth/handedness correlates approach in nonhuman primates (e.g., N=7-15 in capuchin monkeys: Phillips & Sherwood, 2005 Behav Neurosci; Phillips & Thompson 2013 Am J Primatolol; N=63 in baboons: Margiotoudi et al., 2019 Cortex; N=60 in chimps: Dadda et al 2006). We referred to this literature in the introduction and discussion section.

**Replicates**

- You should report how often each experiment was performed
- You should include a definition of biological versus technical replication
- The data obtained should be provided and sufficient information should be provided to indicate the number of independent biological and/or technical replicates
- If you encountered any outliers, you should describe how these were handled
- Criteria for exclusion/inclusion of data should be clearly stated
- High-throughput sequence data should be uploaded before submission, with a private link for reviewers provided (these are available from both GEO and ArrayExpress)

Please outline where this information can be found within the submission (e.g., sections or figure legends), or explain why this information doesn’t apply to your submission:

This information could be found in the method section

The data are available in “OSF Storage” with the identifier DOI 10.17605/OSF.IO/DPXS5. <https://osf.io/dpxs5/?view_only=f406ad972edd43e485e5e4076bae0f78> (manuscript under “data availability” section)

**Statistical reporting**

- Statistical analysis methods should be described and justified
- Raw data should be presented in figures whenever informative to do so (typically when N per group is less than 10)
- For each experiment, you should identify the statistical tests used, exact values of N, definitions of center, methods of multiple test correction, and dispersion and precision measures (e.g., mean, median, SD, SEM, confidence intervals; and, for the major substantive results, a measure of effect size (e.g., Pearson's r, Cohen's d)
- Report exact p-values wherever possible alongside the summary statistics and 95% confidence intervals. These should be reported for all key questions and not only when the p-value is less than 0.05.

Please outline where this information can be found within the submission (e.g., sections or figure legends), or explain why this information doesn’t apply to your submission:

All this information can be found within the submission in the “Methods, statistical analysis” section, “Results” section and every figure legend.

(For large datasets, or papers with a very large number of statistical tests, you may upload a single table file with tests, Ns, etc., with reference to sections in the manuscript.)

**Group allocation**

- Indicate how samples were allocated into experimental groups (in the case of clinical studies, please specify allocation to treatment method); if randomization was used, please also state if restricted randomization was applied
- Indicate if masking was used during group allocation, data collection and/or data analysis

Please outline where this information can be found within the submission (e.g., sections or figure legends), or explain why this information doesn’t apply to your submission:

The way we determined our groups is clearly stated in the “Methods” section of the paper. We added precision in the revision MS about our factors of exclusion:

- lines 262-272: “(…) Out of this sample, were included only subjects which overlaps with both (1) the sample of subjects for which individual measures of handedness for communicative gestures were available (i.e., hand slapping gesture, Meguerditchian & Vauclair, 2006) and (2) the previous sample of 63 subjects (i.e., 35 right-handed and 28 left-handed) reported in Margiotoudi et al. (2019) for which both Central sulcus depth measures and individual measures of handedness for manipulative actions (i.e. the bimanual tube task, Vauclair et al., 2005) were reported. It resulted a total overlap of 50 baboons (29 females and 21 males, mean age (years): M = 12.3, SE = 5.8) who combined thus the both types of measures of handedness (communication versus manipulation) and the depth measures of the two sulci of interest (IA sulcus and Central sulcus) in the two hemispheres of the brain.”

- lines-317-318 “(…) , excluding subjects with less than 5 observations (Mean=25.98, S.E.=3.67).”

- lines 328-330: “A 51th subject, having a HI score of 0 (i.e., no manual bias), could not be classified in either categories and has been thus excluded from the study.”

**Additional data files (“source data”)**

- We encourage you to upload relevant additional data files, such as numerical data that are represented as a graph in a figure, or as a summary table
- Where provided, these should be in the most useful format, and they can be uploaded as “Source data” files linked to a main figure or table
- Include model definition files including the full list of parameters used
- Include code used for data analysis (e.g., R, MatLab)
- Avoid stating that data files are “available upon request”

Please indicate the figures or tables for which source data files have been provided:

-The scripts used for data analysis and the list of subjects are available in “OSF Storage” with the identifier DOI 10.17605/OSF.IO/DPXS5. <https://osf.io/dpxs5/?view_only=f406ad972edd43e485e5e4076bae0f78> (manuscript under “data availability” section)
